# Comparison of cow-side diagnostic tests for subclinical mastitis of dairy cows in Musanze district, Rwanda

**DOI:** 10.4102/jsava.v88i0.1464

**Published:** 2017-06-21

**Authors:** Blaise Iraguha, Humphrey Hamudikuwanda, Borden Mushonga, Erick Kandiwa, Jean P. Mpatswenumugabo

**Affiliations:** 1 Rwanda Dairy Competitiveness Program II, Kigali, Rwanda; 2 ABSTCM Pty. Ltd, Harare, Zimbabwe; 3 Department of Veterinary Medicine, University of Namibia, Namibia; 4 Department of Veterinary Medicine, University of Rwanda, Rwanda

## Abstract

Four subclinical mastitis diagnostic tests (the UdderCheck^®^ test [a lactate dehydrogenase-based test], the California Mastitis Test [CMT], the Draminski^®^ test [a conductivity-based test] and the PortaSCC^®^ test [a portable somatic cell count-based test]) were compared in a study comprising crossbreed dairy cows (*n* = 30) during September and October 2015. Sensitivity and specificity of the CMT, Draminski^®^ and UdderCheck^®^ tests were compared with the PortaSCC^®^ as reference. The CMT, Draminski^®^ and UdderCheck^®^ test results were compared with the results of the PortaSCC^®^ test using kappa statistics. Duplicate quarter milk samples (*n* = 120) were concurrently subjected to the four tests. Sensitivity and specificity were 88.46% and 86.17% (CMT), 78.5% and 81.4% (Draminski^®^) and 64.00% and 78.95% (UdderCheck^®^). The CMT showed substantial agreement (*k* = 0.66), the Draminski^®^ test showed moderate agreement (*k* = 0.48) and the UdderCheck^®^ test showed fair agreement (*k* = 0.37) with the PortaSCC^®^ test and positive likelihood ratios were 6.40, 4.15 and 3.04, respectively. The cow-level subclinical mastitis prevalence was 70%, 60%, 60% and 56.7% for PortaSCC^®^, CMT, Draminski^®^ and UdderCheck^®^ tests, respectively. At udder quarter level, subclinical mastitis prevalence was 20%, 21.67% and 20.83% for PortaSCC^®^, CMT and UdderCheck^®^, respectively. A correlation (*P* < 0.05) and moderate strength of association were found between the four tests used. The study showed that compared to the PortaSCC^®^ test, the CMT was the most preferable option, followed by the Draminski^®^ test, while the UdderCheck^®^ test was the least preferable option for subclinical mastitis screening.

## Introduction

Mastitis is a complex and multifactorial disease characterised by inflammation of the milk producing parenchyma of the udder and is regarded as the most expensive disease of dairy animals (Bogni et al. 2011; Sudhan & Sharma 2010). Mastitis may be either clinical or subclinical (Špakauskas, Klimienė & Matusevičius 2006). The severity of mastitis depends on the nature of the causative pathogen as well as the age, immunological health and lactation status of the cow (Viguier et al. 2009). Subclinical mastitis is the inflammation of the mammary gland that does not create visible changes in the milk or of the udder (Langer et al. 2014). Although the milk appears normal, cows with subclinical intramammary infections (IMI) produce less milk and with compromised quality (Salvador et al. 2014). Clinical mastitis is characterised by visible changes in the udder and in milk (Reddy et al. 2014). Subclinical mastitis can lead to a 10% – 20% decrease in milk production. In addition, it has an undesirable effect on the constituents and nutritional value of the milk, rendering it of low quality and less fit for processing (Fernando, Spahr & Jaster 1985; Iraguha, Hamudikuwanda & Mushonga 2015). As there are no visible abnormalities in the milk, subclinical mastitis requires special diagnostic tests for detection (Bogni et al. 2011; Salvador et al. 2014). The importance of early detection of mastitis, and in particular subclinical mastitis, is critical (Chagunda et al. 2006).

Identification of intramammary pathogens found in milk is the gold standard for the diagnosis of mastitis. However, this is time-consuming, costly and of limited applicability under field conditions (Rodriguez et al. 2009; Viguier et al. 2009). According to the International Dairy Federation (IDF) recommendations, microbiological status of the quarter and the somatic cell count (SCC) are the most common tests to detect changes in the milk because of an inflammatory process (Sudhan & Sharma 2010).

According to Langer et al. (2014), there are several direct and indirect tests that can detect subclinical mastitis. Field tests (cow-side tests) include the California Mastitis Test (CMT), the modified white side test (MWT), the bromothyl blue card test, determination of electrical conductivity, the chloride estimation test, the modified Aulendorfer mastitis probe test (MAMP), inline monitoring of SCC and infrared thermography (Kamphuis et al. 2008). Laboratory tests include N-acetyl-β-D-glucosaminidase (NAGase), enzyme-linked immunosorbent assay (ELISA) (Polat et al. 2010) and acute phase protein determination in milk and in serum (More 2009). Models based on mastitis indicators are now also available (Chagunda et al. 2006). Most of these tests are preferred as screening tests because they are easy to use and they yield rapid as well as satisfactory results (Leslie et al. 2006). Faye and Saleh (2011) have used the CMT, SCC determination and pathogen identification for mastitis diagnosis in Dromedary camels.

According to Langer et al. (2014), there appears to be compatibility between the results of SCCs and the Draminski^®^ test. In their study, the SCC detected 64.4% of subclinical mastitis in cows, while the Draminski^®^ test detected 59.0%. Sharma, Pandey and Sudhan (2010) reported that the sensitivity of the CMT, the sodium lauryl sulphate (SLST) test and the SCC compared to cultural was 86.07%, 74.63% and 88.60%, respectively, while specificity was 59.70%, 17.16% and 97.76% with a percentage accuracy of 75.52%, 51.64% and 91.94%, respectively. Positive predictive values (PPVs) for the same tests were 76.21%, 57.47% and 98.33%, while negative predictive values (NPVs) were 74.07%, 31.08% and 84.52%, respectively. Although pathogen identification in milk samples is the gold standard for diagnosing mastitis (Langer et al. 2014, Rodriguez et al. 2009), Salvador et al. (2014) used SCC (Fossomatic counts) in their study to evaluate the performance of a portable somatic cell counter (PortaSCC^®^). Salvador et al. (2014) reported 94.12% and 87.30% sensitivity and specificity to identify subclinical mastitis using the PortaSCC^®^ test. Based on the PortaSCC^®^ test properties and its capability to rapidly provide results, Salvador et al. (2014) argued that the PortaSCC^®^ test could be used as an alternative for the laboratory-based cell counter in evaluating milk samples from herds in remote areas under Philippine field conditions.

Milk SCC is a diagnostic parameter for subclinical mastitis (International Dairy Federation 1999). A SCC level below 100 000 cells/mL is accepted to represent a healthy quarter. According to some sources (International Dairy Federation 1971; PortaCheck 2011), SCC levels ≥ 200 000 somatic cells per mL of milk are considered to indicate subclinical mastitis (Salvador et al. 2014). Other countries consider SCC levels ≥ 300 000 indicative of subclinical mastitis (Pitkälä et al. 2001). In yet other areas like Europe, New Zealand and Australia, cases of subclinical mastitis were diagnosed when SCC was ≥ 400 000 cells/mL of milk (Hameed, Sender & Korwin-Kossakowska 2007). Still other countries such as Canada and South Africa consider a mastitis case when SCC is ≥ 500 000 cells/mL (Giesecke & Van den Heever 1974; Hogeveen 2005; Sharma, Chhabra & Sindh 2012; Sharma, Singh & Bhadwal 2011; Van den Heever & Turner 1976). Therefore, mastitis should be detected in a reliable and timely manner based on SCC values; otherwise, subclinical mastitis could develop into a clinical disease (Sharma et al. 2011). In this study, a cut-off SCC value of ≥ 500 000 cells/mL was used as in reports from South Africa and Canada. In general, management practises and conditions in Rwanda are similar to those in South Africa even though Musanze district has a high altitude, high precipitation levels and relatively low temperatures.

There is a paucity of information on results that compare various mastitis screening and detection tests; yet mastitis tests are commonly used in Rwanda. The objective of this study was to compare the specificity, sensitivity and PPVs of four commonly used field-based diagnostic tests (PortaSCC^®^, CMT, Draminski^®^ and UdderCheck^®^) for detection of subclinical mastitis in cattle.

## Materials and methods

### Study area

This study was carried out in Musanze district, Northern Province of Rwanda (1°30′6.94″S; 29°37′59.75″E at 1850 m a.s.l.) during September to October 2015. The majority (91%) of the human population in Musanze district is engaged in agriculture and there are three active milk collection centres (MCCs).

There are two wet seasons in Musanze district, the first of which is from February to May and the second from September to November. Average precipitation ranges between 1000 mm and 1200 mm annually. Average temperatures vary between 17.8 °C and 21 °C. Musanze consists of volcanic, lateritic humus-bearing and clayey soils (http://www.musanzedistrict.gov.rw).

### Data collection

A convenience sample of 30 cows from 7 dairy farms was used in this study. The cows were examined by a veterinarian; no clinical signs of disease were detected and the cows were considered to be healthy. Udders and quarters of cows were physically examined to rule out clinical mastitis. All cows were reared in Musanze and were 3–7 years of age. The cows were not tested for any diseases. A duplicate of udder quarter milk samples was tested for the presence of subclinical mastitis using the PortaSCC^®^, CMT, Draminski^®^ and the lactate dehydrogenase-based UdderCheck^®^ tests. Prior to collection of milk samples, the udder and teats were examined visually and then palpated to detect fibrosis, cardinal signs of inflammation, swelling of supra-mammary lymph nodes, visible injury, abscesses, tick infestation and atrophy of the tissue. The udder and teats were cleaned with water and dried using paper towels. The teat orifice and the skin around the teat were wiped with cotton soaked in 70% alcohol. About 5 mL of the first milk was milked into a strip cup to detect clinical mastitis. Then direct milk samples from the teats were systematically subjected to PortaSCC^®^, CMT, LDH-based UdderCheck^®^ and Draminski^®^. For PortaSCC^®^ test, using a pipette, four drops of the sampled milk of each teat were well added to the test strip sample, and then three drops of activator solution were added to the strip. Evaluation was performed after 45 min using a digital reader (PortaCheck 2011). The CMT test was conducted as described by Quinn et al. (1994), where a squirt of milk from each quarter of the udder was placed in each of the four shallow cups of the CMT paddle and an equal amount of the CMT reagent was added, and then gentle circular motion was applied in a horizontal plane. The UdderCheck^®^ test was conducted according to the method described on the UdderCheck website (http://www.uddercheck.com). Milk was squirted onto a test strip and after 2 min was compared to a colour chart as described by the manufacturer. For Draminski^®^ test, about 15 mL of milk was stripped into a Draminski^®^ cup previously disinfected using methylated spirits, and the milk discarded after a reading appeared on the liquid crystal display (LCD) panel of the Draminski^®^ apparatus as described (Dramiński 1989). The process was repeated for each teat, with care being taken to avoid contamination of the teats. At the end of the process, the on/off button was pressed again, and the LCD displayed readings for the four teats/quarters. The readings were then recorded for each cow. Interpretation of results was based on inter-quarter variations, as described (Dramiński 1989).

Results were recorded for individual udder quarters, right front (RF), right hind (RH), left front (LF) and left hind (LH) for the PortaSCC^®^, CMT and UdderCheck^®^ tests considering that a quarter presenting one case of subclinical mastitis was considered as subclinical mastitis positive, whereas results for Draminski^®^ was interpreted and recorded based on inter-quarter variations, as described (Dramiński 1989).

### Data analysis

In this study, a cut-off SCC count of ≥ 500 000 cells/mL was used to indicate subclinical mastitis.

A two-by-two table reflecting the results of the CMT and UdderCheck^®^ tests was generated. The tests were compared for their ability to detect the prevalence of subclinical mastitis using Chi-square analysis and strength of association tested using Cramer’s V statistic. Where there were significant associations, further comparisons of test pairs were conducted using Chi-square analysis. The results of the PortaSCC^®^ test were used as the reference (previously validated by Salvador et al. [2014]) in the calculation of the test properties of the CMT and UdderCheck^®^ tests. Sensitivity and specificity of CMT and UdderCheck^®^ and their respective confidence intervals (CIs) were calculated using mid-P 95% CI. The agreement between the results of the two tests was evaluated using kappa statistics. The interpretation of the kappa results was based on the proposal of Landis and Koch (1977). Kappa statistics and their interpretation are as follows: poor (< 0.00), slight (0.00–0.20), fair (0.21–0.40), moderate (0.41–0.60), substantial (0.61–80), almost perfect (0.81–1.00). The sensitivity, specificity, disease prevalence, likelihood ratios and the predictive values of the CMT and UdderCheck^®^ tests were calculated and compared with those of the PortaSCC^®^ test (used as reference test). For the Draminski^®^ test, interpretation of results was based on inter-quarter variations, as described (Dramiński 1989).

## Results

Cow-level subclinical mastitis prevalence was 70%, 60%, 60% and 56.7% for the PortaSCC^®^, CMT, Draminski^®^ and UdderCheck^®^ tests, respectively (Table 1), while quarter subclinical mastitis prevalence was 20%, 21.67% and 20.83% for PortaSCC^®^, CMT and UdderCheck^®^, respectively (Tables 2 and 3). The prevalence of subclinical mastitis did not differ (*P* < 0.05) among the tests (Tables 4 and 5). The prevalence of subclinical mastitis detected by the CMT and UdderCheck^®^ tests showed significant, moderately strong associations with that from the PortaSCC^®^ test (Table 4), while the Draminski^®^ test had significant, moderate strength of association with the CMT and UdderCheck^®^ tests (Table 5). Subclinical mastitis prevalence, which was determined using the CMT test, also had a significant (*P* = 0.004), moderate strength (Cramer’s *V* = 0.522) association with that from the UdderCheck^®^ test.

**TABLE 1:** Mastitis prevalence using different tests.

MCC	Number of farms	Number of cows tested	Number of mastitis +ve cows	% mastitis +ve
PortaSCC	7	30	21	70.0
Draminski^®^	7	30	18	60.0
UdderCheck^®^	7	30	17	56.7
CMT	7	30	18	60.0
**Total**	**7**	**120**	**-**	**62.0†**

CMT, California Mastitis Test; MCC, milk collection centre.

† , 95% confidence limits were 51.5% – 71.0%.

**TABLE 2:** Cross tabulation of California Mastitis Test and PortaSCC^®^ results.

Outcomes	Teats with subclinical mastitis as confirmed by the PortaSCC^®^ test		Predictive values
	Condition positive	Condition negative	
CMT outcome	True positive: 23	False positive: 13	Positive predictive value: 63.89%
CMT outcome	False negative: 3	True negative: 81	Negative predictive value: 96.43%
Sensitivity and specificity	Sensitivity: 88.46%	Specificity: 86.17%	-

CMT, California Mastitis Test.

**TABLE 3:** Cross tabulation of UdderCheck^®^ and PortaSCC^®^ results.

Outcomes	Teats with subclinical mastitis as confirmed by the PortaSCC^®^ test		Predictive values
	Condition positive	Condition negative	
UdderCheck^®^ outcome	True positive: 16	False positive: 20	Positive predictive value: 44.44%
UdderCheck^®^ outcome	False negative: 9	True negative: 75	Negative predictive value: 89.29%
Sensitivity and specificity	Sensitivity: 64.00%	Specificity: 78.95%	-

**TABLE 4:** Association between the PortaSCC^®^ test and other tests used to screen quarter milk samples for the presence of subclinical mastitis.

Tests	Chi-square value	*df*	Significance level (*p*-value)	Strength of association – Cramer’s V
CMT	7.646	1	0.006	0.505
Draminski^®^	7.646	1	0.006	0.505
UdderCheck^®^	6.456	1	0.0062	0.492

CMT, California Mastitis Test; *df*, degree of freedom.

**TABLE 5:** Association between Draminski^®^ and other tests used to screen quarter milk samples for subclinical mastitis.

Tests	Chi-square value	*df*	Significance level (*p*)	Strength of association – Cramer’s V
CMT	10.208	1	0.001	0.583
UdderCheck^®^	8.167	1	0.004	0.522

CMT, California Mastitis Test; *df*, degree of freedom.

A two–by-two table comparing results of the PortaSCC^®^ test and the Draminski^®^ test showed an unweighted kappa value of 0.48 (± 0.07); a positive likelihood ratio of 4.15 and a negative likelihood ratio of 0.31.

Further statistical analysis of the results in Table 2 showed an unweighted kappa value of 0.66 (± 0.06); a positive likelihood ratio of 6.40, a negative likelihood ratio of 0.13 and a disease prevalence of 21.67% for the CMT at quarter level.

Statistical analysis of the results in Table 3 showed also an unweighted kappa value of 0.37 (± 0.07); a positive likelihood ratio of 3.04, a negative likelihood ratio of 0.46 and a disease prevalence of 20.83% for the UdderCheck^®^ at quarter level.

## Ethical considerations

This study was conducted considering animal welfare, animal well-being and animal rights.

## Discussion

The PortaSCC^®^ test has been successfully used as a reference for comparison with other tests under field conditions by Salvador et al. (2014) under Philippine field conditions. Sargeant et al. (2001) reported that CMT could be used in dairy herd monitoring programmes as a screening test to detect cows with IMI caused by major pathogens. Barbosa et al. (2002) reported that the SCC and CMT were highly correlated for the diagnosis of subclinical mastitis.

Our study revealed that the sensitivities and specificity of the CMT compared more favourably with the PortaSCC test than the UdderCheck^®^ test and would be the best replacement test to use in the absence of the PortaSCC^®^ test. According to Parikh et al. (2008), higher sensitivity for a test means that the test is better able to diagnose disease in animals with that disease while higher specificity for a test means that the test is better able to diagnose disease-free animals.

The CMT showed substantial agreement (*k* = 0.66) with the PortaSCC^®^ test, while the Draminski^®^ test showed moderate agreement (*k* = 0.48) and the UdderCheck^®^ test showed fair agreement (*k* = 0.37) with the PortaSCC^®^ test.

The higher *k*-value when the PortaSCC^®^ test and the CMT tests are compared means that the agreement between these tests is because of intrinsic traits of the tests rather than because of chance. Sharma, Maiti and Pandey (2008) stated that a positive CMT test reaction depends on the concentration of somatic cells in the milk.

There was a substantial agreement between the PortaSCC^®^ and the CMT tests in the detection of subclinical mastitis, indicated by high levels of sensitivity, specificity and positive and negative predicted values. This, however, was not found to be true for the UdderCheck^®^ test. The PortaSCC^®^ test and the CMT respond to the presence of somatic cells in milk, whereas the UdderCheck^®^ test responds to the presence of the lactate dehydrogenase enzyme that is released even before the subclinical mastitis stage. However, while the UdderCheck^®^ test did not have similar high sensitivity, specificity, PPV and NPV compared to the PortaSCC^®^ test, it may still detect subclinical mastitis at expectable levels and hence could still be an acceptable screening test for subclinical mastitis (*P* < 0.05) (PortaCheck 2013).

Positive likelihood ratios were 6.40, 4.15 and 3.04 for CMT, Draminski^®^ and UdderCheck^®^ tests with the PortaSCC^®^ test. According to McGee (2002), PPV close to 1 means that the test in question is not useful for the disease in question.

Obtained cow-level subclinical mastitis prevalence was 70%, 60%, 60% and 56.7% for the PortaSCC^®^, CMT, Draminski^®^ and UdderCheck^®^ tests, respectively. The prevalence of cow subclinical mastitis detected by the tests ranged from 56.7% to 70% and this was within the range reported by Chatikobo (2010) and closely similar to 52% reported by Iraguha et al. (2015) in Nyagatare district both using the Draminski^®^ test. Although it was higher than those reported in studies from Ethiopia (34% – 46%) (Ayano et al. 2013; Abera et al. 2010) and Bangladesh 44.8% (Rahman et al. 2009); it was lower than 75.9% reported by Karimuribo et al. (2008) from Tanzania. Udder quarter subclinical mastitis prevalence was 20%, 21.67% and 20.83% for PortaSCC^®^, CMT and UdderCheck^®^ tests, respectively. These prevalences are close to the 19.1% obtained, using CMT, by Sanotharan, Pagthinathan and Nafees (2016) in Sri Lanka but lower than the 34.8% obtained by Mekibib et al. (2010) in Ethiopia.

Our study showed that the CMT was the next most preferable option after the PortaSCC^®^ test followed by the Draminski^®^ test. The UdderCheck^®^ test was shown to be the least preferable option for screening the cows in Musanze for subclinical mastitis.

The cow-level subclinical mastitis prevalence results obtained from the four tests showed a moderate association with each other, indicating that all the tests could be used for detection of subclinical mastitis.

## Conclusion

Our results showed an overall cow-level subclinical mastitis prevalence of 62% in the study area. The results from this study showed significant, moderately strong association (correlation) among the PortaSCC^®^, CMT, Draminski^®^ and UdderCheck^®^ tests. These tests can therefore be used for screening for subclinical mastitis. Our study showed that the CMT was the next most preferable option after the PortaSCC^®^ test and the UdderCheck^®^ test was the least preferable option for screening the cows in Musanze for subclinical mastitis.

## References

[CIT0001] Abera M., Demiel B., Aragawl K., Regassa F. & Regassa M.A, 2010, ‘Isolation and identification of *Staphylococcus aureus* from bovine mastitic milk and their drug resistance patterns in Adama town, Ethiopia’, *Journal of Veterinary Medicine and Animal Health* 2(3), 29–34.

[CIT0002] Ayano A.A., Hiriko F., Simyalew A.M. & Yohannes A, 2013, ‘Prevalence of subclinical mastitis in lactating cows in selected commercial dairy farms of Holeta district’, *Journal of Veterinary Medicine and Animal Health* 5(3), 67–72. 10.5897/JVMAH12.056

[CIT0003] Barbosa C.B., Benadetti E., Ribeiro S.C., Guimaraes E.C. & Ribeiro S.C, 2002, ‘The relationship between somatic cell count (SCC) and result of the California Mastitis Test (CMT) to diagnose bovine mastitis’, *Bioscience Journal* 18(1), 93–102.

[CIT0004] Bogni C., Odierno L., Raspanti C., Giraudo J., Larriestra A., Reinoso E. et al., 2011, ‘War against mastitis: Current concepts on controlling bovine mastitis pathogens’, in Méndez-Vilas A. (ed.), *Science against microbial pathogens: Communicating current research and technological advances*, pp. 483–494, Rio Cuarto, Science against microbial pathogens: Communicating current research and technological advances, Argentina.

[CIT0005] Chagunda M.G.G., Friggens N.C., Rasmussen M.D. & Larsen T, 2006, ‘A model for detection of individual cow mastitis based on an indicator measured in milk’, *Journal of Dairy Science* 89(8), 2980–2998. 10.3168/jds.S0022-0302(06)72571-1 16840614

[CIT0006] Chatikobo P, 2010, ‘Mastitis control for quality milk’, *Dairy Mail Africa* 5(1), 8–11.

[CIT0007] Dramiński J, 1989, ‘The relationship of milk electrical resistance and health of the udder, Cooperation of Polish Agricultural Universities, Poland’, viewed 17 September 2015, from http://www.draminski.com

[CIT0008] Faye S.S.B. & Saleh S.K, 2011, ‘Detection of subclinical mastitis in dromedary camels (Camelus dromedaries) using somatic cell counts, California Mastitis Test and udder pathogen’, *Emiratus Journal of Food and Agriculture* 23(1), 48–58. 10.9755/ejfa.v23i1.5312

[CIT0009] Fernando R.S., Spahr S.L. & Jaster E.H, 1985, ‘Comparison of electrical conductivity of milk with other indirect methods for detection of subclinical mastitis’, *Journal of Dairy Science* 68(2), 449–456. 10.3168/jds.S0022-0302(85)80844-4 3989085

[CIT0010] Giesecke W.H. & Van den Heever L.W, 1974, ‘The diagnosis of bovine mastitis with particular reference to subclinical mastitis, a critical review of relevant literature’, *Onderstepoort Journal of Veterinary Research* 41(4), 169–212. 4620089

[CIT0011] Hameed K.G.A., Sender G. & Korwin-Kossakowska A, 2007, ‘Public health hazard due to mastitis in dairy cows’, *Animal Science Papers and Reports* 25(2), 73–85.

[CIT0012] Hogeveen H. (ed.), 2005, ‘Mastitis in dairy production: Current knowledge and future solutions. Book Type: Conference Proceedings’, ISBN: 9789076998701, 10.3920/978-90-8686-550-5

[CIT0013] International Dairy Federation, 1971, *A monograph on bovine mastitis*, International Dairy Federation, Brussels, Belgium.

[CIT0014] International Dairy Federation, 1999, ‘Suggested interpretation of mastitis terminology’, *Bulletin of the International Dairy Federation* 338, 3–26.

[CIT0015] Iraguha B., Hamudikuwanda H. & Mushonga B, 2015, ‘Bovine mastitis prevalence and associated risk factors in dairy cows in Nyagatare District, Rwanda’, *Journal of the South African Veterinary Association* 86(1), 1–6. 10.4102/jsava.v86i1.1228 PMC6138073 26244583

[CIT0016] Kamphuis C., Sherlock R., Jago J., Mein G. & Hogeveen H, 2008, ‘Automatic detection of clinical mastitis is improved by in-line monitoring of somatic cell count’, *Journal of Dairy Science* 91(12), 4560–4570. 10.3168/jds.2008-1160 19038931

[CIT0017] Karimuribo E.D., Fitzpatrick J.L., Swai E.S., Bell C., Bryant M.J., Ogden N.H. et al., 2008, ‘Prevalence of subclinical mastitis and associated risk factors in smallholder dairy cows in Tanzania’, *Veterinary Record* 163(1), 16–21. 10.1136/vr.163.1.16 18603630

[CIT0018] Landis J.R. & Koch G.G, 1977, ‘The measurement of observer agreement for categorical data’, *International Biometric Society* 33(1), 159–174. 10.2307/2529310 843571

[CIT0019] Langer A., Sharma S., Sharma N.K. & Nauriyal D.S, 2014, ‘Comparative efficacy of different mastitis markers for diagnosis of sub-clinical mastitis in cows’, *International Journal of Applied Sciences and Biotechnology* 2(2), 121–125. 10.3126/ijasbt.v2i2.10191

[CIT0020] Leslie K., Barratt K., Petersson C. & Bashiri A, 2006, ‘An evaluation of the PortaSCC^®^ test as a measure of Udder Health Status Dairy Cows (an excerpt from a technical report)’, viewed 20 August 2015, from http://allindiadairy.com/Atricles-2011-12/An%20Evaluation%20of%20the%20PortaSCC%20%2060-61.pdf

[CIT0021] McGee S, 2002, ‘Simplifying likelihood ratios’, *Journal of General Internal Medicine* 17(8), 647–650. 10.1046/j.1525-1497.2002.10750.x PMC1495095 12213147

[CIT0022] Mekibib B., Furgasa M., Abunna F., Megersa B. & Regassa A, 2010, ‘Bovine mastitis: Prevalence, risk factors and major pathogens in dairy farms of Holeta Town, Central Ethiopia’, *Veterinary World* 3(9), 397–403. 10.5455/vetworld.2010.397-403

[CIT0023] More S.J, 2009, ‘Global trends in milk quality: Implications for the Irish dairy industry’, *Irish Veterinary Journal* 62 (Suppl 4), S5 10.1186/2046-0481-62-S4-S5 22081986 PMC3339351

[CIT0024] Parikh R., Mathai A., Parikh S., Sekhar G.C. & Thomas R., 2008, ‘Understanding and using sensitivity, specificity and predictive values’, *Indian Journal of Ophthalmology* 56(1), 45–50. PMID:18158403 10.4103/0301-4738.37595 PMC2636062

[CIT0025] Pitkälä A., Haveri M., Pyörälä S., Myllys V. & Honkanen-Buzalski T, 2001, ‘Bovine mastitis in Finland 2001 – Prevalence, distribution of bacteria, and antimicrobial resistance’, *Journal of Dairy Science* 87(8), 2433–2441. 10.3168/jds.S0022-0302(04)73366-4 15328265

[CIT0026] Polat B., Colak A., Cengiz M., Yanmaz L.E., Oral H., Bastan A. et al., 2010, ‘Sensitivity and specificity of infrared thermography in detection of subclinical mastitis in dairy cows’, *Journal of Dairy Science* 93(8), 3525–3532. 10.3168/jds.2009-2807 20655420

[CIT0027] PortaCheck, 2011, *On-farm testing solutions for the dairy industry*, Moorestown, NJ, viewed 23 September 2015, from http://www.portacheck.com

[CIT0028] PortaCheck, 2013, *Quick test for udder infection*, NJ, viewed 03 July 2015, from http://www.uddercheck.com

[CIT0029] Quinn P.J., Carter M.E., Marke B.K. & Carter G.R, 1994, *Clinical veterinary microbiology*, 1st edn, Mosby-Year book Europe, Ltd., Wolfe, Spain.

[CIT0030] Rahman M.A., Bhuiyan M.M.U., Kamal M.M. & Shamsuddin M, 2009, ‘Prevalence and risk factors of mastitis in dairy cows’, *Bangladesh Veterinarian* 26(2), 54–60. 10.3329/bvet.v26i2.4951

[CIT0031] Reddy B.S.S., Kumari K.N., Reddy Y.R., Reddy M.V.B. & Reddy B.S, 2014, ‘Comparison of different diagnostic tests in subclinical mastitis in dairy cattle’, *International Journal of Veterinary Science* 3(4), 224–228.

[CIT0032] Rodriguez A.C.O., Cassoli L.D., Machado P.F., Ruegg P.L. & Reinemann D.J, 2009, ‘Short communication: Evaluation of an on-farm test to estimate somatic cell count’, *Journal of Dairy Science* 92(3), 811–1315. 10.3168/jds.2008-1216 19233792

[CIT0033] Salvador R.T., Soliven R.L., Balagan E.J.Y., Abes N.S., Gutierrez C.A. & Mingala C.N, 2014, ‘Evaluation of a portable somatic cell counter in the diagnosis of bubaline subclinical mastitis’, *Thai Journal of Agricultural Science* 47(4), 205–209.

[CIT0034] Sanotharan N., Pagthinathan M. & Nafees M.S.M, 2016, ‘Prevalence of bovine subclinical mastitis and its association with bacteria and risk factors in milking cows of Batticaloa District in Sri Lanka’, *International Journal of Scientific Research and Innovative Technology* 3(6), 2313–3759.

[CIT0035] Sargeant J.M., Leslie K.E., Shirley J.E., Pulkrabek B.J. & Lim G.H, 2001, ‘Sensitivity and specificity of somatic cell count and California mastitis test for identifying intramammary infection in early lactation’, *Journal of Dairy Science* 84(9), 2018–2024. 10.3168/jds.S0022-0302(01)74645-0 11573781

[CIT0036] Sharma N., Maiti S.K. & Pandey V, 2008, ‘Sensitivity of indirect tests in the detection of subclinical mastitis in buffaloes’, *Veterinary Practitioner* 9(1), 29–31.

[CIT0037] Sharma N., Pandey V. & Sudhan N.A, 2010, ‘Comparison of some indirect screening tests for detection of subclinical mastitis in dairy cows’, *Bulgarian Journal of Veterinary Medicine* 13(2), 98–103.

[CIT0038] Sharma N., Singh N.K. & Bhadwal M.S, 2011, ‘Relationship of somatic cell count and mastitis: An overview’, *Asian-Australian. Journal of Animal Science* 24(3), 429–438. 10.5713/ajas.2011.10233

[CIT0039] Sharma P.A., Chhabra R. & Sindh N, 2012, ‘Prevalence of subclinical mastitis in cows: Its etiology and antibiogram’, *Indian Journal of Animal Research* 46(4), 348–353.

[CIT0040] Špakauskas V., Klimienė I. & Matusevičius A, 2006, ‘A comparison of indirect methods for diagnosis of subclinical mastitis in lactating dairy cows’, *Veterinarski Arhiv* 76(2), 101–109.

[CIT0041] Sudhan N.A. & Sharma N, 2010, ‘Mastitis – An important production disease of dairy animals, *SMVS’* (Serva Manav Vikas Samiti) *Dairy Year Book*, Ghaziabad, Uttar Pradesh, India’, viewed 17 May 2016, from http://www.smvsdairyyearbook.com

[CIT0042] Van den Heever L.W. & Turner G.V.S, 1976, ‘Subclinical bovine mastitis: Comparison of results of two sets of diagnostic criteria’, *Journal of the South African Veterinary Association* 47(4), 263–264. 1018294

[CIT0043] Viguier C., Arora S., Gilmartin N., Welbeck K. & O’Kennedy R, 2009, ‘Mastitis detection: Current trends and future perspectives’, *Trends in Biotechnology* 27(8), 486–493. 10.1016/j.tibtech.2009.05.004 19616330

